# Muscarinic receptors in the rat ovary are involved in follicular development but not in steroid secretion

**DOI:** 10.14814/phy2.15474

**Published:** 2022-11-02

**Authors:** Fernanda C. Cuevas, Daniela Bastias, Constanza Alanis, Agustin Benitez, Valentina Squicciarini, Raul Riquelme, Pia Sessenhausen, Artur Mayerhofer, Hernan E. Lara

**Affiliations:** ^1^ Centre for Neurobiochemical Studies in Neuroendocrine Diseases, Laboratory of Neurobiochemistry, Faculty of Chemistry and Pharmaceutical Sciences Universidad de Chile Santiago Chile; ^2^ Biomedical Center, Cell Biology, Anatomy III, Faculty of Medicine Ludwig Maximilian University of Munich Martinsried Germany

**Keywords:** acetylcholine, atresia, follicular development, Huperzine‐A, muscarinic receptors

## Abstract

Acetylcholine (ACh) may be involved in the regulation of ovarian functions. A previous systemic study in rats showed that a 4‐week, intrabursal local delivery of the ACh‐esterase blocker Huperzine‐A increased intraovarian ACh levels and changed ovarian follicular development, as evidenced by increased healthy antral follicle numbers and corpora lutea, as well as enhanced fertility. To further characterize the ovarian cholinergic system in the rat, we studied whether innervation may contribute to intraovarian ACh. We explored the cellular distribution of three muscarinic receptors (MRs; M1, M3, and M5), analyzed the involvement of MRs in ovarian steroidogenesis, and examined their roles in ovarian follicular development in normal conditions and in animals exposed to stressful conditions by employing the muscarinic antagonist, atropine. Denervation studies decreased ovarian norepinephrine, but ovarian ACh was not affected, evidencing a local, nonneuronal source of ACh. M1 was located on granulosa cells (GCs), especially in large antral follicles. M5 was associated with the ovarian vascular system and only traces of M3 were found. Ex vivo ovary organo‐typic incubations showed that the MR agonist Carbachol did not modify steroid production or expression of steroid biosynthetic enzymes. Intrabursal, in vivo application of atropine (an MR antagonist) for 4 weeks, however, increased atresia of the secondary follicles. The results support the existence of an intraovarian cholinergic system in the rat ovary, located mainly in follicular GCs, which is not involved in steroid production but rather via MRs exerts trophic functions and regulates follicular atresia.

## Graphical abstract

Role of Acetylcholine in steroid biosynthesis and follicular development in the rat ovary. The follicles of the rat ovary do not receive a direct extraovarian cholinergic innervation. It mainly originated from an intraovarian source of Acetylcholine (a). This ACh does not regulate biosynthesis of steroid hormones and also it is not manifested as an increase in the efflux of steroid hormones in vitro (b). The main effect is on follicular development as was demonstrated by Urra et al. (Sci Report, 2016, **6**: p. 30129) or by the administration of the cholinergic muscarinic agonist Carbachol (Benitez et al., Front Endocrinol, 2021, **12**: p. 636600). The presence of Atropine, a well‐known muscarinic antagonist, increased atresia of secondary follicles (c), a process that is antagonized by cold stress, a process that increases ovarian acetylcholine (Riquelme et al., J Neuroendocrinol, 2020: p. e12914).

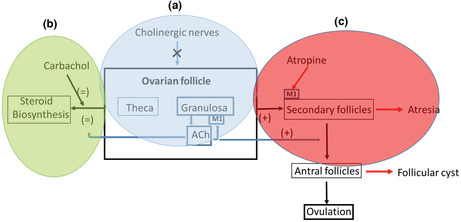

## 1 INTRODUCTION

The ovary of the rat is regulated by hormones and, although less well known, also by sympathetic and parasympathetic neurotransmitters. Sympathetic nerve endings release norepinephrine (NE) and communicate with the ovary via the superior ovarian nerve and the ovarian plexus nerve. Branches of the superior ovarian nerve are located around the follicles and regulate steroid secretion and follicular development, while branches of the ovarian plexus nerve mainly innervate blood vessels (Gerendai et al., 2002; Lawrence Jr. & Burden, 1980).

In contrast to sympathetic nerves, only a few parasympathetic nerves, that is, branches of the vagus nerve, enter the ovarian hilar region and are usually associated with blood vessels (Burden & Lawrence Jr., 1978). This view is based on acetylcholinesterase‐positive nerves, which are present as perivascular plexuses in rats (Lawrence Jr. et al., 1978). It has not been demonstrated that vagal nerve branches reach ovarian follicular walls, as is the case for sympathetic nerve fibers.

Species differences in ovarian parasympathetic innervation patterns may exist. For example, very few such fibers are reported in the chicken ovary (Unsicker et al., 1983) and no such fibers are reported in the human ovary (Mayerhofer & Kunz, 2005). Despite the apparent lack of innervation, the prototype parasympathetic neurotransmitter acetylcholine (ACh) was detected in human ovarian cells, that is, luteinizing granulosa cells (GCs), but also in bovine luteal cells (Al‐Zi'abi et al., 2009). In rodent immortalized GCs, follicular stimulating hormone (FSH) increases ACh (Mayerhofer et al., 2006). Thus, as described for a variety of cells throughout the body, especially epithelial cells, ovarian ACh is produced by GCs (Mayerhofer & Kunz, 2005; Wessler & Kirkpatrick, 2017).

The roles of ACh in the ovary, in general, are not well known but GCs from different species is a target of ACh. Several in vivo and in vitro studies demonstrated that muscarinic ACh receptors (MRs) (Wessler & Kirkpatrick, 2017) are present in GCs. Their molecular identities, however, remain to be fully explored (Batra et al., 1993). Elucidation of this point has, among others, been hampered because the specificity of many ACh receptor antibodies has been questioned (Rommel et al., 2015). That ovarian MRs are, however, functional was shown, for example, by the antagonizing actions of the MR blocker atropine. Such actions include trophic, growth‐promoting influences of ACh on GCs in bovine and human ovary‐derived cells (Al‐Zi'abi et al., 2009; Mayerhofer & Kunz, 2005). Other consequences of ACh and its analog, carbachol (Cch), include changes in intracellular pH (Li et al., 1992), rapid increases in intracellular Ca^2+^ levels (Mayerhofer et al., 1992), activation of ion channels (Kunz et al., 2002), and disruption of gap junctional communication (Fritz et al., 2002).

In neuronal tissues, actions of ACh are primarily regulated by the enzymatic breakdown of the molecule. Similar mechanisms may be assumed in the ovary (Prado et al., 2013) and involve two enzymes, namely butyrylcholinesterase (BuChE) and acetylcholinesterase (AChE). Both are present in the ovary and human follicular fluid (Blohberger et al., 2015; Urra et al., 2016). Indeed, several AChE isoforms, derived from alternative splicing, are present in the human and nonhuman primate ovary and in GCs. The specific AChE inhibitor huperzine A (HupA) (Wang et al., 2006) was used in cultures of human GCs and in cultured nonhuman primate follicles (Blohberger et al., 2015; Du et al., 2018). The results, for example, enhanced survival of follicles, further indicated the trophic actions assigned to elevated ACh (Wessler & Kirkpatrick, 2017).

Based on such in vitro studies, we recently performed a systemic study in rats (Urra et al., 2016). As we detected AChE in the rat follicular compartment, we examined the consequences of a 4‐week intrabursal local delivery of HupA on ovary function, follicular growth, and fertility in the rat. We found that HupA increased intraovarian ACh levels and changed ovarian follicular development, as evidenced by increased numbers of healthy antral follicles, corpora lutea, and enhanced fertility success in the rat (Urra et al., 2016). It appears possible that these changes are direct consequences of higher ACh levels and activation of specific cholinergic receptors in the ovary (Benitez et al., 2021; Riquelme et al., 2020), thus the hypothesis to be tested is that ACh from local origin acts on ovarian follicles through muscarinic receptors present in ovarian cells of the follicle.

To elucidate the hypothesis, we

- examined whether ovarian innervation contributes to intraovarian ACh;
- explored the cellular distribution of MR focusing on M1, M3, and M5 for which validated antibodies exist;
- analyzed the function of MR in ovarian steroidogenesis, and
- studied the role of the receptors in ovarian follicular development under normal and under stressful conditions with or without the muscarinic antagonist, atropine.

The results obtained support an intraovarian cholinergic system in the rat ovary, located in follicular granulosa cells. This system is not involved in steroid production but rather exerts trophic functions and prevents follicular atresia.

## 2 METHODS

### 2.1 Animals

Sprague–Dawley rats from our facilities, weighing 250–300 g, were maintained at 20°C in a 12‐h light 12‐h darkness cycle. Water and food were available ad libitum. The estrus cycling activity of the rats was monitored via daily vaginal smears and observed under a light microscope as previously described (Fernandois et al., 2012; Marcondes et al., 2002; Piquer et al., 2022). The number of cycles was estimated as the regular passage from proestrus (P) to estrus (E) followed by diestrus (D). At the end of the experiments, the rats were euthanized by decapitation, and the ovaries and plasma were collected. Decapitation was performed by a specialized worker according to the AVMA Guidelines for the Euthanasia of Animals (2020 Edition) (AVMA, 2020). The study was also approved by the Bioethics Committee of the Faculty of Chemistry and Pharmaceutical Sciences at the University of Chile (protocol number CBE2017‐05 for HL) and complied with the national guidelines (CONICYT Guide for the Care and Use of Laboratory Animals). Further ovaries (*n* = 11) were obtained from 3‐month‐old Sprague–Dawley rats from the animal facility at the BMC, Planegg (Germany) and used for organ‐specific incubations (Table 1).

**TABLE 1:** Distribution of the rats for the study

	*n*	Age at end	Days of treatment	Ovary studies	Ovarian morphology
In vitro steroid secretion					
Stress 28 days	5	90	28	Steroid secretion	—
Control	5	90	28	Steroid secretion	—
Stimulation of steroid enzymes					
Carbachol	7	90	6 h‐Inc	Steroid enzymes	—
HupA	4	90	6 h‐Inc	Steroid enzymes	—
Biochemical studies					
Stress 28 days	5	90	28	WB, mRNA	—
Control	5	90	28	WB, mRNA	—
Cold stress 28 days/atropine					
28d (sham operated)	4	90	28	Morphometry	Yes
28d (+ 10 μM atropine)	4	90	28	Morphometry	Yes
28d (stress)	4	90	28	Morphometry	Yes
28d (stress+10 μM atropine)	4	90	28	Morphometry	Yes
Denervation studies					
Surgical denervation					
Sham‐operated rats	4	90	21	NE, ACh	—
Denervated rats (SON or PN)	8	90	21	NE, ACh	—
Chemical denervation					
Sham (saline injected)	3	90	21	NE, ACh	—
Neonatal guanethidine‐treated rats	3	90	21	NE, ACh	—

*Note*: It is shown that the physiological procedures done, the total number of animals used (*n*), and its distribution for each of the experiments presented in the work. It is also shown the age of the rats, the treatment for each ovary, and the biochemical determination.

Abbreviations: ON, ovarian nerve; SON, superior ovarian nerve.

### 2.2 Denervation studies

a.‐ Transection of the superior ovarian nerve (SON) and plexus nerve (PN). The SON was selected for transection because it carries sympathetic fibers that predominantly innervate the endocrine component of the ovary, in contrast to the PN, which mainly innervates the ovarian vasculature (Lawrence Jr. et al., 1978; Lawrence Jr. & Burden, 1980). Four adult rats were anesthetized with an intraperitoneal (i.p.) dose of ketamine 60 mg/kg/xylazine 10 mg/kg solution under aseptic conditions. The bilateral ovaries were exposed through a dorsal incision, and the SON (accompanied by the ovarian ligament) was sectioned with a microcautery. The PN accompanying the blood vessels sectioned with a microcautery, as described (Lara et al., 2002; Lara, Hill, et al., 1990). Rats were euthanized 21 days after surgery. All experiments were carried out 21 days after the surgical procedure (when ovarian NA was lower (Lara et al., 2002)).

b.‐ Neonatal treatment with Guanethidine (GD) was performed as previously described (Lara et al., 2002; Lara, McDonald, et al., 1990). We used six neonatal rats. On postnatal day 7, three rats have treated with guanethidine monosulfate solution (Sigma Aldrich, MO). It was administered i.p. at a dose of 50 mg/kg, 5 days/week, for 3 weeks starting on postnatal day 7. The drug was dissolved in saline, the pH adjusted to 7.4, and the solution sterilized by passage through a 0.22‐μm filter. Control rats (Sham) received an equivalent volume of saline (Lara, McDonald, et al., 1990). After treatment, GD and Sham rats were euthanized at 90 days old.

### 2.3 Cold stress procedure

Twenty adult female rats were randomly assigned to either a nonstress group (control group, *n* = 10) or a stress group (*n* = 10). Stress exposure was designed according to Dorfman et al. (Dorfman et al., 2003) and Bernuci et al. (Bernuci et al., 2013). Briefly, cold stress promotes ovarian morphological alterations related to a polycystic ovary syndrome condition through activation of the ovarian sympathetic nerves (Bernuci et al., 2013). Rats used for the cold stress procedure were transported, in their cages, to a 4°C cold room and remained there for 3 h each day, Monday–Friday, for 4 weeks. Control rats were moved to a location near the cold room and returned to the animal room after 3 h. At the end of the stress protocol period, control and stressed rats were euthanized, and serum was collected and stored at −80°C. The ovaries of five controls and five stressed rats were used for in vitro steroid secretion studies. One ovary of the remaining rats (control and stressed) was fixed for immunohistochemistry studies and the other ovary was divided in half. One half was used for western blot analysis and the other half was stored at −80°C for molecular studies, as detailed below.

### 2.4 In vivo atropine administration studies

Sixteen adult female rats were randomly assigned to either a sham group (*n* = 4), atropine group (*n* = 4), stress (*n* = 4), or stress + atropine group (*n* = 4). Animals were anesthetized with an intramuscular (i.m.) dose of ketamine 60 mg/kg/xylazine 10 mg/kg solution under aseptic conditions. To eliminate the possible contribution of the contralateral ovary to steroidogenesis, all control, stressed, and atropine‐treated animals were unilateral ovariectomized at the moment of the minipump implant (Squicciarini et al., 2018). Minipump implantation was performed as previously reported (Lara et al., 2000). Briefly, we did a small incision (1 cm long) through the skin and localized the left or right lumbar aponeurosis, separated the muscle, and got access to the ovary. Either in the sham group or in the experimental group, one ovary (usually the right ovary) was ligated to isolate it from blood vessels and the ovary was dissected and saved for other experiments. In the left ovary, we exposed the bursa and performed a microsurgery incision to insert a cannula (SILASTIC 0.64 mm ID × 1.19 mm OD CAT 508‐003; Dow Corning Corp) connected to the miniosmotic pump implanted underneath the skin. The cannula was kept in place to the bursa wall with a drop of surgery glue (Histoacryl, B.Braun Surgical, S.A) and sutures attached to the ipsilateral uterine horn. Delivering a flow rate of 2.5 μl/h, these pumps remain operational for 28 days. Animals of the atropine group were implanted with osmotic minipumps for intraovarian atropine delivery at an ovarian concentration of 10 μM (catalog number 212385‐M, Calbiochem, Sigma Chemicals). After 28 days, rats were euthanized, and the ovary and trunk blood were collected for analysis. Ovaries were fixed with Bouin's fluid for embedding, sectioning, and morphometric analyses.

### 2.5 Ovarian organ incubation studies

#### 2.5.1 In vitro ovarian steroid secretion

Five cold‐stressed and five control rats were euthanized and both ovaries were removed through an anterior incision in the midline of the abdomen. Ovaries were halved (2 ovaries = 4 halves per animal), and each half was incubated for 3 h at 37°C in 1.0 ml of Krebs‐bicarbonate‐albumin (NaCl 118.6 mM, KCl 4.7 mM, KH2PO4 1.2 mM, ascorbic acid 100 μg/mL, NaHCO3 0.15 M, CaCl2 25 mM, albumin 0.1 mg/mL, an glucose 11.2 μg/mL) under 95% oxygen and 5% CO2. For each rat, one‐half ovary was incubated in Krebs‐bicarbonate buffer, and the second was incubated with an additional 100 μM of carbachol (Cch) as a stimulus for MR (catalog number 212385‐M, Sigma Chemicals, St Louis, MO, USA). The third half ovary was incubated in Krebs‐bicarbonate buffer with 10 μM of atropine (At), a muscarinic antagonist (catalog number A0257‐10 g, Sigma Chemicals), + 100 μM of Cch. After 3 h of incubation, the incubation media were collected to measure basal and induced secretion of testosterone, progesterone, and estradiol using the EIA kit, following the manufacturer's instructions. The catalog number of the test kits were 11‐TESHU‐E01, 11‐ESTHU‐E01, and 11‐PROHU‐E01 for testosterone, E2, and P4, respectively (Alpco Diagnostic).

#### 2.5.2 Detection of steroidogenic enzymes

Incubations of additional rat ovaries (*n* = 11) for 6 h were performed following a slightly different protocol. In brief, after extraction, ovaries were halved and then either mechanically disrupted using sterile syringe needles and submersed in a medium (*n* = 3), or directly placed on agar blocks at the interface air/medium (*n* = 8). Tissues placed in multi‐well plates were then placed on a prewarmed orbit shaker (100 rpm) at 37°C, 95% oxygen, and 5% CO_2_ and incubated for 6 h in either DMEM/F12 containing 10 μM Cch (*n* = 7) or HupA (*n* = 4), or DMEM/F12 containing PBS, as solvent control. After incubation, ovarian tissues were frozen in liquid nitrogen and stored at −80°C. Disruption of the ovarian tissues and RNA extraction were performed using the RNeasy Plus Universal Mini Kit (Qiagen), following the manufacturer's protocol. The concentration of isolated RNA was then measured using a spectrophotometer (NanoDrop 2000c, Thermo Fisher Scientific Inc.). After the conversion of RNA into cDNA via reverse transcription (SuperScriptTM II Reverse Transcriptase; Invitrogen), quantitative RT‐PCR (LightCycler 96® System, Roche Diagnostics) was performed for the detection of StAR, CYP11A1, and CYP19A1.

### 2.6 Immunohistochemistry of M1, M3, and M5 receptors

Immunohistochemistry (IHC) was performed on 6 μm slices from rat ovaries fixed and embedded in paraffin, as previously described (Riquelme et al., 2019; Squicciarini et al., 2018). Antigen retrieval was done by microwaving for 10 min in buffer TRIS‐EDTA pH 9.0, and the samples were then washed three times in PBS. Samples were incubated for 30 min in 3% hydrogen peroxide with 10% methanol to block endogenous peroxidases. The antibodies used were all validated by the manufacturer: rabbit polyclonal anti‐M1 antibody (AMR‐001, Alomone Labs) in a 1:1000 dilution, rabbit polyclonal anti‐M5 antibody (AMR‐005, Alomone Labs) in a 1:1000 dilution, and rabbit polyclonal anti‐M3 antibody (AMR‐006, Alomone Labs) at 1:800 dilution, adding a permeabilization step with 10% Triton X100 for M1 and M3 immunostaining before antigen retrieval. Antibody incubations were performed overnight in PBS supplemented with 5% normal horse serum. On the second day, slices were washed in PBS and incubated with the secondary biotinylated antibody (1:200) for 1 h. The signal was detected using the Vectastain ABC kit (VectorLabs) according to the manufacturer's instructions. Preadsorption was performed as previously described following the manufacturer's instructions.

### 2.7 PCR studies

RNA was extracted by the method described by Chomczynski and Sacchi (Chomczynski & Sacchi, 1987) from the half ovary (control and stress). For the first‐strand cDNA synthesis, we used SUPERSCRIPT II (Invitrogen; 200 units) in a 20 μl reaction volume with 1 μg of total RNA. The primers used were from the published work of (Iismaa et al., 2000), and a BLAST analysis was performed to determine the specificity of the sequences. The sequences of the primers for real‐time PCR are M1: F 5’‐TCTGAGACACCAGGCAAAGG‐3 and R 5’‐CTTGACTGTATTTGGGGAGC‐3′ (gene blast M16406), M3: F 5′‐ ACAGGCAGTTCTCGAAGCT‐3′ and R 5’‐ACGGTAGCTTGGTAGAG‐3′ (gene blast M16407), and M5: F 5′‐ AAACAGTTGTGAACACCCG‐3′ and R 5′‐ CTCTTTGACCAGAACCATTC‐3′ (gene blast M22926).

The PCR reaction mix contained 10 μl of Brilliant II SYBR Green QPCR Master Mix (Agilent Technologies, Inc.); 0.25 μM of each GAPDH primer, 0.1 μM of each MR primer, 2 μl of cDNA, and sterile water for a final volume of 20 μl. PCR reactions were performed using the IQ5 real‐time thermocycler (BioRad) under the following conditions: 94°C for 20 s, 60°C for 20 s for MR (60°C for GAPDH), 78°C for 20 s, and a final extension of 72°C for 10 min. All samples for RT‐qPCR analysis were run in triplicate and a mean value was used for the determination of mRNA levels. Relative quantification of MR mRNA levels was performed using the ratio between MR and GAPDH mRNA levels.

Further qPCR studies were performed to examine the effect of Cch and HupA on steroid biosynthetic enzymes. The sequences of the primers are StAR: F 5´‐GGGCATACTCAACAACCAGGA‐3′ and R 5´‐GTCTAGCAGCACCTCCAGTC‐3′, CYP11A1: F 5′‐ GAGATCCCTTCCCCTGGTGA and R 5′‐ ACTGACTCCATGTTGCCCAG‐3′, and CYP19A1: F 5′‐ TGGATGGGGATTGGAAGTGC‐3′ and R: 5′‐ CTTGCTGCCGAATCTGGAGA‐3′. GAPDH was used for normalization. The PCR reaction mix contained 6.25 μl SYBR Green (QuantiFast SYBR Green PCR kit; Qiagen), 3.33 μM of each primer, and 4 μl of cDNA. For these PCR reactions, the LightCycler 96® System (Roche Diagnostics) was used. Since results from incubations with mechanically dispersed ovaries did not differ from the ones placed on agar blocks at the interface air/medium, the results are presented together in the case of Cch.

### 2.8 Morphometric analysis

The ovaries were fixed in Bouin's fluid, embedded in paraffin, cut into 6‐mm sections, and stained with hematoxylin and eosin. Morphometric analyses of whole ovaries were performed according to the method described by Hirshfield (Hirshfield & Midgley Jr., 1978) with modifications (Lara et al., 2000), using *n* = 4 ovaries for each of the experimental groups. We used the following classification: primordial follicles had one oocyte surrounded by flattened GCs. Primary follicles had one layer of cuboidal GCs, and secondary follicles had no antral cavity but two or more layers of GCs. Antral follicles were those with more than three healthy GC layers, the antrum, and a clearly visible nucleus of the oocyte. Atretic follicles had more than 5% of the cells with pyknotic nuclei in the largest cross‐section and exhibited shrinkage and occasional breakdown of the germinal vesicle.

### 2.9 Western blot of MR: M1, M3, and M5

Detection of ovarian M1, M3, and M5 and the internal control β‐actin by western blot was performed, following separation by SDS–PAGE on 10% polyacrylamide gels under reducing conditions. The proteins were transferred to nitrocellulose membranes (pore size: 0.45 μm; Schleicher & Schuell), blocked with 5% milk for 1 h, and probed with either rabbit polyclonal anti‐M1 antibody (AMR‐001, Alomone Labs) in a 1:1000 dilution, rabbit polyclonal anti‐M5 antibody (AMR‐005, Alomone Labs) in a 1:500 dilution, overnight or in a 1:10,000 dilution of mouse monoclonal anti‐β‐actin (A1978, Sigma–Aldrich Co.) for 1h. The antibody complexes were detected using goat antirabbit IgG Fc (HRP) (ab97200, Abcam, Inc.) or horse antimouse IgG Fc (HRP) (BA‐2000, Vector Lab) both at 1:10000, and for chemiluminescence, an EZ‐ECL Enhanced Chemiluminescence Detection Kit (Biological Industries, KBH, Israel) was used. Chemiluminescence was captured using a G‐Box Syngene system (Syngene Headquarters). Band intensity was quantified by ImageJ software (Rasband, 1997–2014), and values obtained were normalized to β‐actin. Each protein sample detection was assessed in three independent experiments.

### 2.10 Norepinephrine (NE) and acetylcholine (ACh) determination

- Quantification of NE levels was performed using the competitive norepinephrine ELISA kit Research® (IMMUSMOL), according to the manufacturer's instructions. Tissue was homogenized in 0.01 N HCl, 1 mM EDTA, and 4 mM sodium metabisulfite. NE from the homogenate was extracted by affinity gel, acylated, and then derivatized enzymatically. The antigen was bound to the solid phase of the microtiter plate. The derivatized standards, controls, and samples, as well as the solid‐phase‐bound analyte, competed for a fixed number of antiserum binding sites. The antibody bound to the solid phase was detected using an antirabbit IgG‐peroxidase conjugate and tetramethylbenzidine (TMB) as a substrate. The reaction was monitored at 450 nm. The sensitivity was 2 pg/ml, and the intra‐ and interassay variability were 8.4% and 8.0%, respectively. The cross‐reactivity found was 0.14% for adrenaline and 1.8% for dopamine.
- For ACh determination, the ovary was homogenized in 10 volumes of PBS in ice. ACh was determined in the homogenate using the Amplex® ACh/AChE assay kit (Invitrogen), according to the instructions recommended by the provider, as previously described (Riquelme et al., 2019; Urra et al., 2016). The results represent the total amount of ACh in μmol per ovary. The minimal detectable value for ACh was 0.3 μM (range, 0.3 μM to 100 μM).

### 2.11 Steroid hormones measurements

Steroids hormones released to the medium during incubation of the ovaries with cholinergic and/or antagonist and serum concentrations of steroid hormones testosterone, estradiol (E2), and progesterone (P4) were determined via EIA, following the manufacturer's instructions. The catalog numbers of the test kits were 11‐TESHU‐E01, 11‐ESTHU‐E01, and 11‐PROHU‐E01 for testosterone, E2, and P4, respectively (Alpco Diagnostic). Intra‐ and interassay variations were less than 5% for E2, less than 6% for testosterone, and less than 5% for P4. The minimal detectable values were 10, 0.02, and 0.1 ng/ML, respectively.

### 2.12 Statistics

The data are expressed as the mean ± SEM. Statistical analyses were performed using GraphPad Prism 6 (GraphPad Software). To test for significant differences between the two groups, we used the Student's *t*‐test, paired two‐tailed t test, and the Mann–Whitney *U* test when data did not present normality. To test for significant differences between more than two groups, we used one‐way ANOVA, followed by the Newman–Keuls post hoc test to check for the differences between pairs of data.

To select the number of animals for each experiment, we calculated the minimum number of animals needed according to the variability of the experimental procedures and the intrinsic variation between individual animals. The minimum number of animals was calculated with the following equation (Zar, 1984):

$$n=\frac{2{\left(\mathrm{Z\alpha}+\mathrm{Z\beta}\right)}^{2}\times S^{2}}{d},$$

where *n* is the number of animals for each condition, *S* represents standard deviation, *d* is the difference needed to obtain statistical significance, Zα is the probability of type I error (significance), and Zβ is the probability of type II error (power). In the experiments, we proposed α = 0.05 for the probability of obtaining a statistically significant difference by chance, β = 0.3 for the minimum probability of finding a difference between the populations if one exists, 0.2 for the intrapopulation variation, and *d* = 0.11 for the smallest difference in the population. Thus, we obtained *n* = 4.5. Therefore, to obtain a statistically significant difference of *p* < 0.05, we needed to use four or five animals per study group.

## 3 RESULTS

### 3.1 Consequences of surgical or chemical denervation on intraovarian NE and ACh levels

**FIGURE 1:**
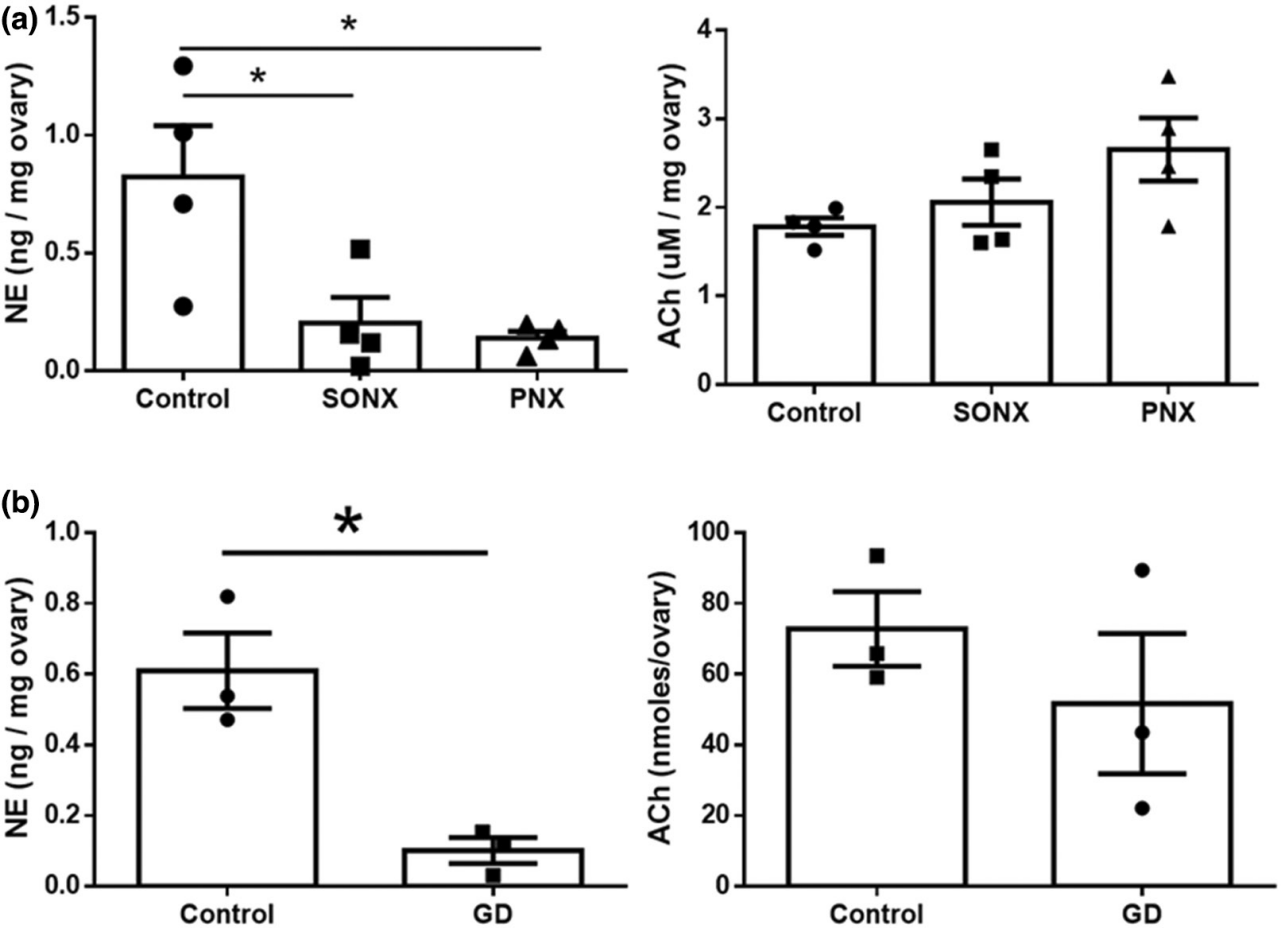
Effect of two different techniques of denervation in the content of ovarian norepinephrine (NE) or acetylcholine (ACh). (a) Effect of surgical denervation of the superior ovarian nerve (SONX) or the plexus nerve (PNX) on the concentration of NE and ACh in the ovary (*n* = 4). (b) Effect of neonatal treatment with guanethidine (GD) on both neurotransmitters (*n* = 3). Results are expressed as mean ± S.E.M. *p < 0.05, unpaired Student's *t* test.

Figure 1 depicts the consequences of surgical sectioning of the SON or PN on ovarian ACh and NE levels (after 21 days). NE decreased upon both procedures but ACh ovarian levels were not significantly decreased (Figure 1a). Similar results were found after GD treatment, that is, a 90% decrease in NE but no significant difference in ACh (Figure 1b). Hence, ovarian ACh is mainly derived from intraovarian sources.

### 3.2 Intraovarian distribution of MR

**FIGURE 2:**
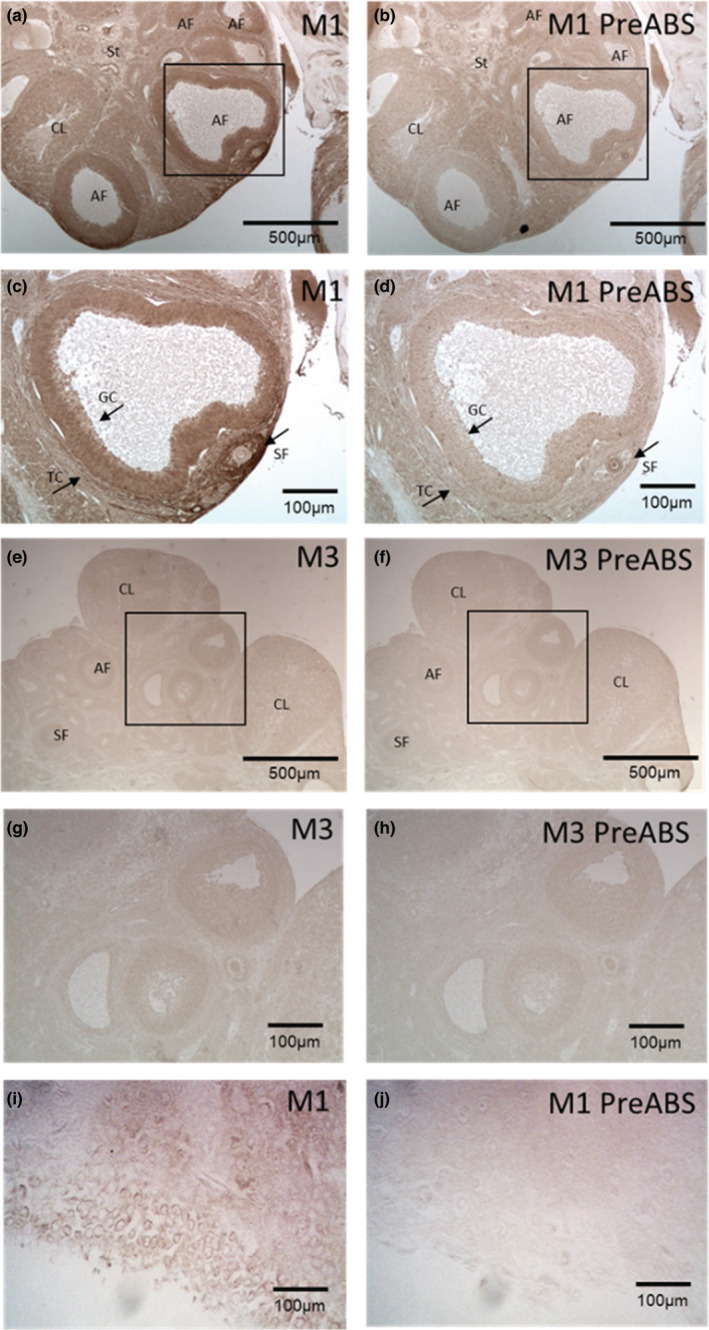
Immunohistochemical detection of M1 and M3 in the rat ovary. M1 is mainly detected in antral and preovulatory follicles but is also present in smaller follicles (a: 10×, c: 40×). M3 is not detected in any ovarian structure (e: 10×, g: 40×). Preabsorbed (Pre‐ABS) antibody figures of M1 (b: 10×, d: 40×) and M3 (f: 10×, h: 40×) are shown as a negative control. I is a positive control for the M1, neurons in rat hippocampus, and J is the negative control without primary antibody for I. AF, Antral follicle; CG, Granulosa cells; CL, Corpora lutea; SF, Secondary follicle; St, Stromal tissue; TC, Theca cells.

**FIGURE 3:**
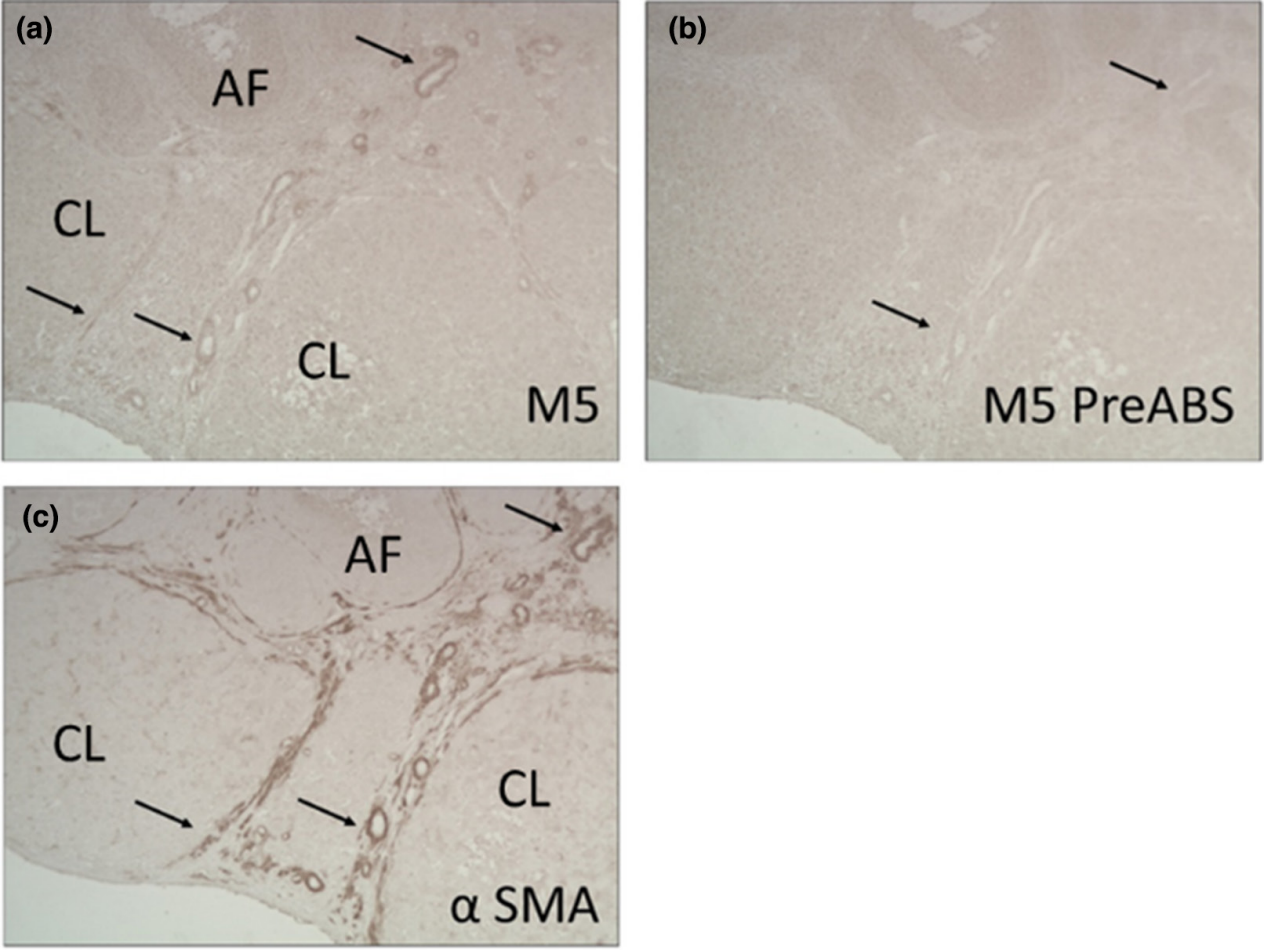
Immunohistochemical detection of M5 in the rat ovary. M5 is detected in small blood vessels (arrows) (a). As a positive control of blood vessels, we used an anti‐αSMA actin antibody (c). Preabsorbed (Pre‐ABS) antibody of M5 (b) is shown as a negative control. Arrows show the immunopositive signals for both M5 and αSMA in the same structures. AF, Antral follicles; CL, Corpora lutea.

Immunostaining of rat ovarian sections showed M1 receptors in GCs of preovulatory follicles and in secondary follicles (SF) (Figure 2a,c). Staining was also detected in theca cells and the corpus luteum, but the intensity was lower than in preovulatory structures (Figure 2a,c). Rat hippocampus served as a positive control (Figure I, J). Specific staining for M3 in ovary was, in contrast, not detectable (Figure 2e–h). M5 staining was exclusively enriched at small and round‐shaped structures localized at the stromal region of the ovary (Figure 3a–b). They also stained positive when we used the α‐smooth muscle actin (αSMA) antibody (Figure 3c), which marks vascular smooth muscle cells of blood vessels.

### 3.3 The effect of in vitro muscarinic stimulation on steroid production

**FIGURE 4:**
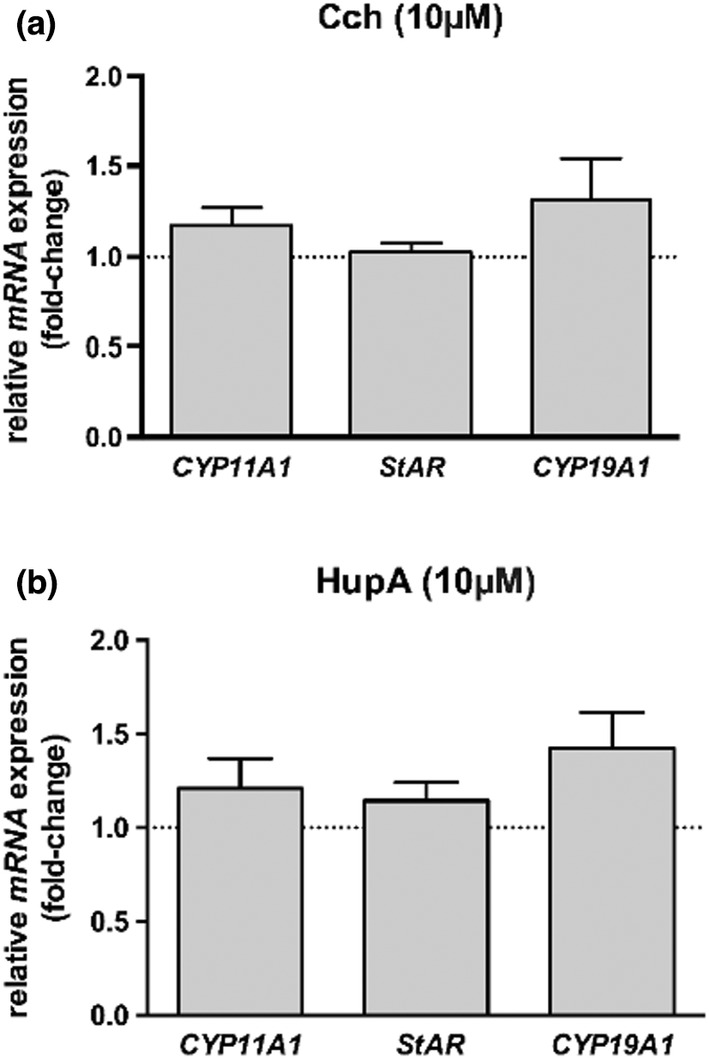
Changes in expression of three steroidogenic enzymes incubated with cholinergic modulators. Ovaries from 3‐month‐old Sprague–Dawley rats were incubated for 6 h with carbachol (Cch) 10 μM or Huperzine a (HupA) 10 μM. mRNA qRT‐PCR quantification was performed to examine the influence of (a) Cch (*n* = 7) and (b) HupA (*n* = 4) on expression levels of *CYP11A1*, *StAR*, and *CYP19A1*. No significant differences were observed. The graphs represent the mean ± SEM normalized to solvent controls (indicated by the dotted lines).

**FIGURE 5:**
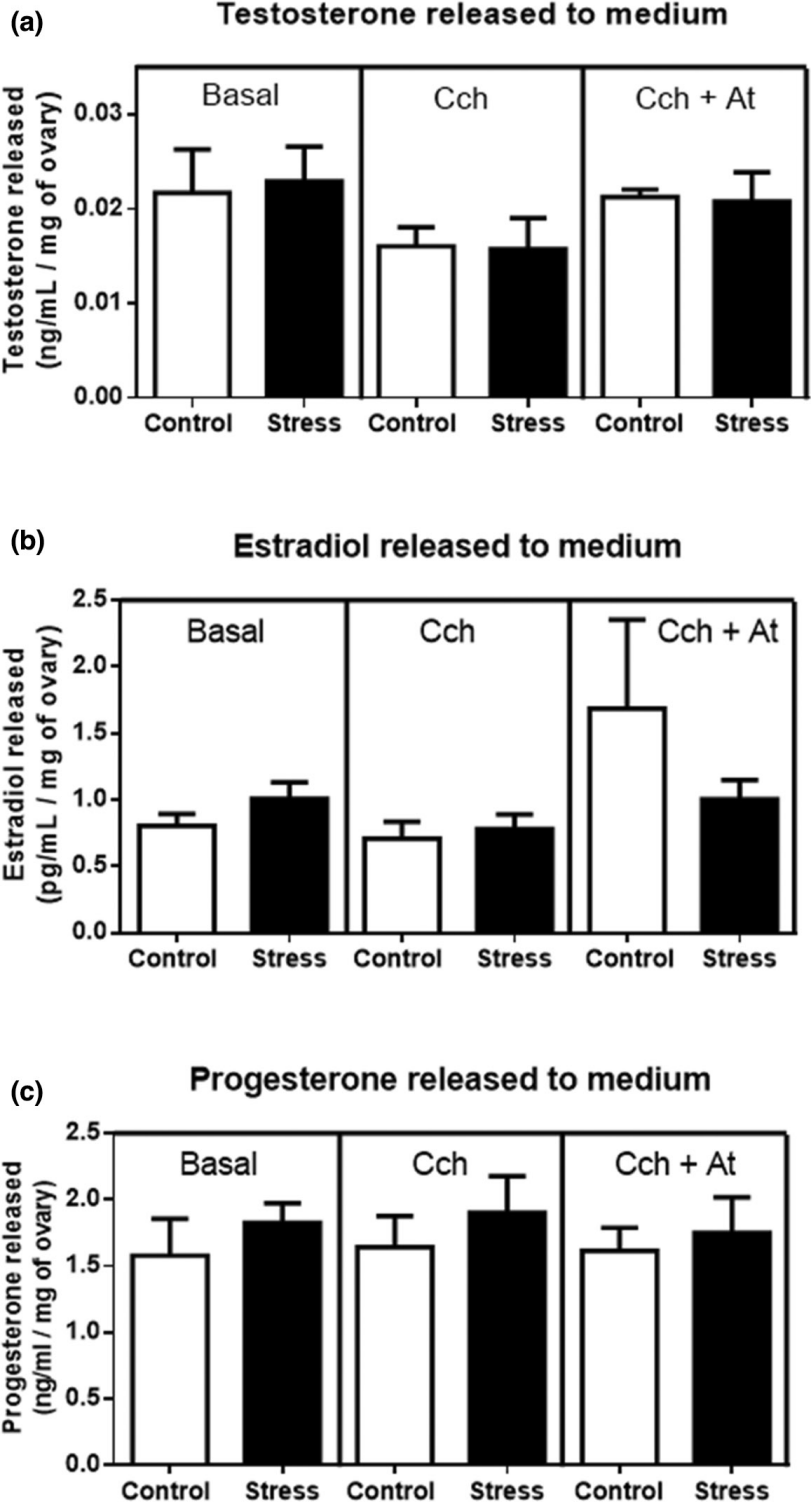
Steroid measurements of testosterone (a), estradiol (b), and progesterone accumulation in a medium under basal conditions, carbachol (100 μM), and atropine (10 μM). The ovaries of each rat were divided and incubated for 3 h to measure steroid secretion. The groups correspond to control and stress. Results are expressed as mean ± S.E.M (*n* = 5).

We incubated ovaries (from control animals) for 6 h (Figure 4). Quantitative RT‐PCR was performed to examine the influence of (A) Carbachol (Cch; *n* = 7) and (B) HupA (*n* = 4) on expression levels of *CYP11A1*, *StAR*, and *CYP19A1*. The treatment with Cch (MR agonist) or with HupA (AChE‐blocker) did not change the expression of the steroid biosynthetic enzymes studied. To further study the possible effect of ACh on steroidogenesis and to exclude any systemic contribution to this process, we performed short‐timed incubations (3 h) with ovaries isolated from control and cold‐stressed animals in the presence of carbachol with or without atropine. Levels of Testosterone (T), Estradiol (E2), and Progesterone (P4) released to the incubation media were measured. We did not detect any significant changes between ovaries from control and in vivo‐stressed animals (Figure 5). When we incubated ovaries from control or 28 days cold‐stressed rats with carbachol and/or atropine, we were not able to detect changes with respect to the basal condition (Figure 5). Taken together, this indicates that local ACh via MR most likely does not participate in steroidogenic processes in the ovary.

### 3.4 The effect of stress on MR in the ovary

**FIGURE 6:**
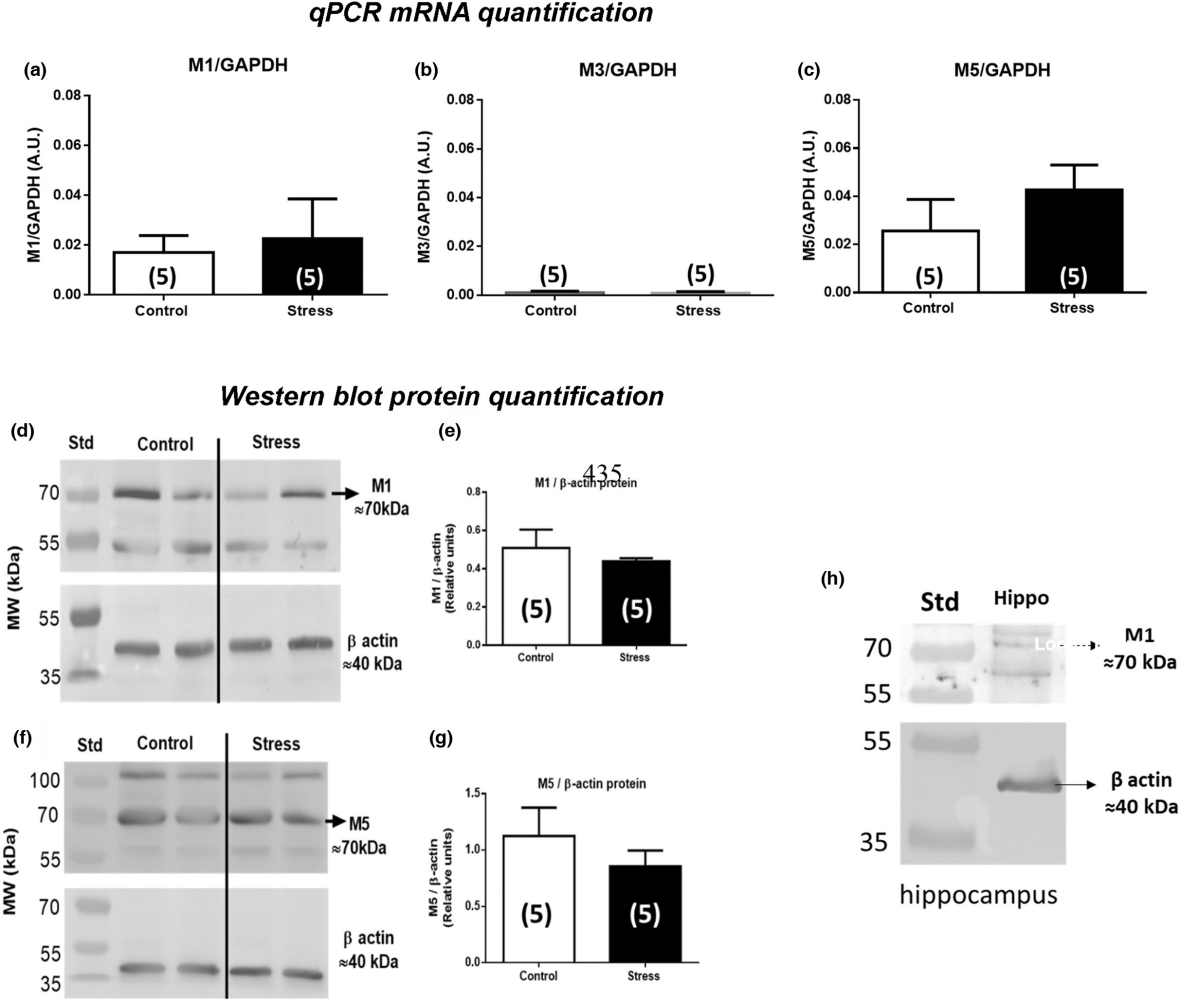
Expression of *M1* (a), *M3* (b), and *M5* (c) normalized to GAPDH mRNA. d, e, and f show the results for western blot analysis. Representative membranes of M1 (d) and M5 (f) are shown. The bar chart shows the quantification of protein levels of M1 (d) and M5 (f) compared with β‐Actin control in each condition. A tissue sample from the hippocampus was used as a control (h). Results are expressed as mean ± S.E.M (*n* = 5).

Previous data described an increase in ACh concentration in the ovary of rats exposed to sympathetic stress (Riquelme et al., 2019), that is, when rats were exposed to chronic cold stress for 28 days. Figure 6 shows the results of measurements of mRNA (A‐C) and protein levels of M1, M3, and M5 receptors (D‐E) by RT‐qPCR and western blot, respectively, in ovary samples of control and stressed rats. Rat hippocampus served as a positive control. As expected by the results of the immunohistochemistry analyses, the M3 subtype was not detectable by western blot analysis (data not shown). M1 and M5 mRNA were found in the ovary and the corresponding proteins were demonstrated in western blots. Neither mRNA nor protein levels changed in the ovary of rats exposed to chronic cold stress for 28 days.

### 3.5 Effect of blocking the muscarinic receptors on follicular development of the ovary

**FIGURE 7:**
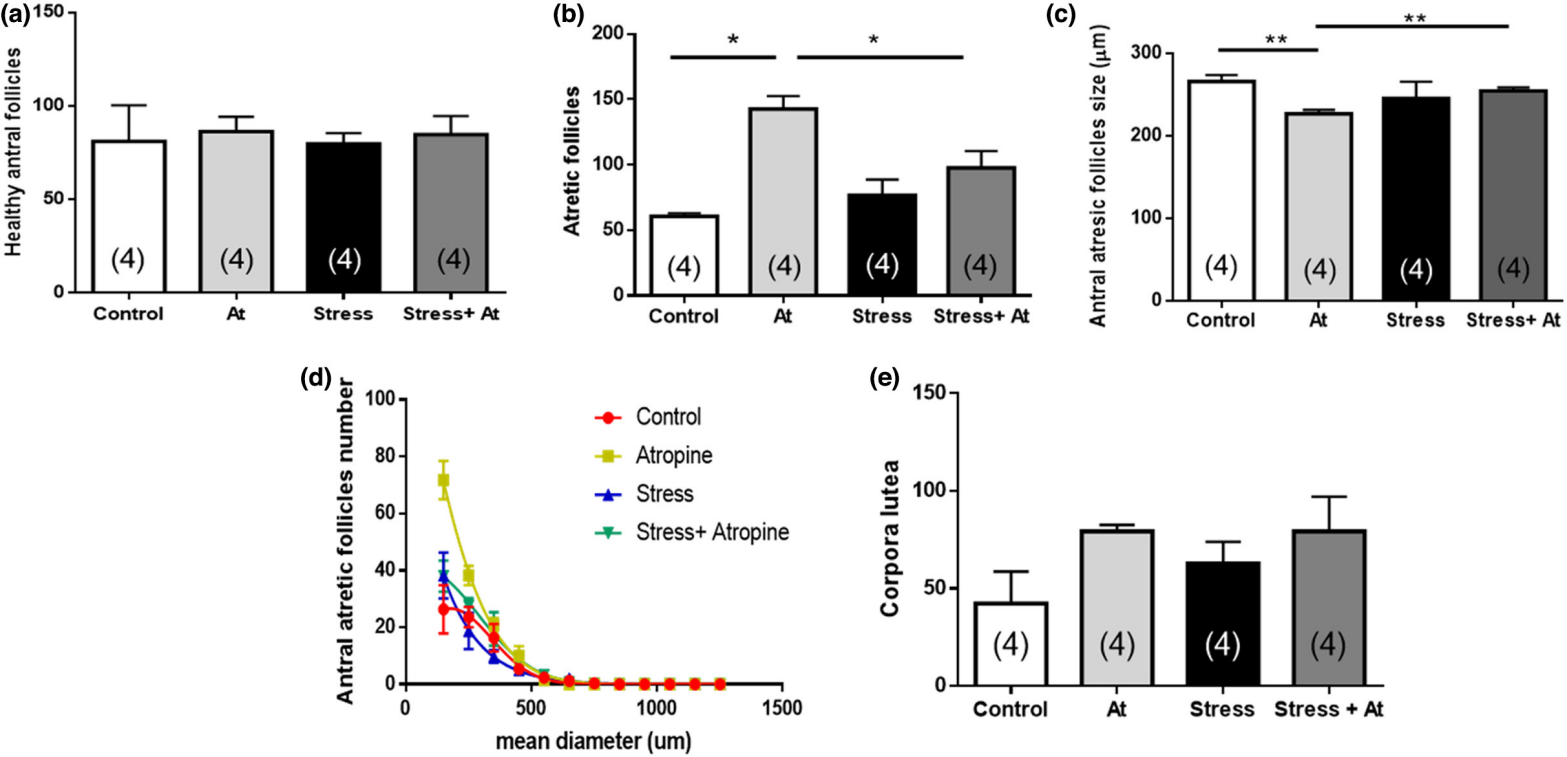
Morphometric analysis of ovaries after in vivo intra‐bursal exposure with atropine (at) 10 μM for 28 days. Control animals (control) were exposed to sham surgery; in the At group, animals were exposed to atropine 100 μM locally delivered to the ovary using an osmotic minipump. The stress group (stress) was exposed to cold stress for 28 days and the (stress + At) group was exposed to stress and received at 10 μM for 28 days. Morphometric analysis in the ovaries of the number of healthy antral follicles (a), atretic antral follicles (b) size of antral follicles (c), the size distribution of atretic follicles (d), and the number of corpora lutea (e) are presented. Graphs correspond to mean ± SEM (*n* = 4). **p < 0.01, *p < 0.05, unpaired Student's *t* test.

Figure 7 shows the results of the morphometric evaluation of ovarian sections, namely changes in the numbers of follicles and CL after chronic atropine application under control and cold‐stress conditions. No effect of atropine on the number of healthy antral follicles was found, neither in controls nor after 28 days of cold stress (Figure 7a). Atropine application, however, significantly increased the atresia of antral follicles, especially under control conditions and, in part, under stress conditions (Figure 7b). Atropine treatment further resulted in a significant decrease in the size of small atretic antral follicles (Figure 7c,d). There was no significant change in the number of corpora lutea.

## 4 DISCUSSION

A previous systemic study in rats (Urra et al., 2016) examined the consequences of a 4‐week intrabursal local delivery of HupA. This treatment elevated intraovarian ACh, increased numbers of healthy antral follicles, corpora lutea, and enhanced fertility success in female rats (Urra et al., 2016). These changes could conceivably be a direct consequence of higher intraovarian ACh levels and activation of cholinergic receptors in the ovary (Benitez et al., 2021; Riquelme et al., 2020).

In the present study, we studied some open questions, namely whether ovarian innervation may contribute to intraovarian ACh, in addition to intraovarian production, and examined the presence and intraovarian localization of three of the five known MR subtypes (M1/M3/M5). We also examined ACh/MR participation in steroid biosynthesis and in follicular development.

The results of denervation studies showed reduced NE levels, but unchanged intraovarian ACh levels, arguing for a mainly intra‐ovarian production of ACh. M1 and M5 were found in the rat ovary. M1 was associated with GCs from preantral and antral follicles and corpora lutea. M5 was localized to ovarian blood vessels. Muscarinic stimulation was not linked to the regulation of steroid production from the ovary both in vitro and in vivo. Rather, it was associated with follicular development, indicated by the action of atropine to increase follicular atresia. Hence, the results show that the local ovarian ACh system is involved in the regulation of follicular dynamics in the rat.

Evidence for M1, M3, and M5 was provided in rat, macaque, and in human granulosa cells obtained from in vitro fertilization (Cruz et al., 2015; Kozlowska et al., 2014; Mayerhofer & Fritz, 2002; Urra et al., 2016). MRs in the ovary, in general, were previously associated with ovulation (Cruz et al., 2015), steroid biosynthesis (Flores et al., 2011), and compensatory hypertrophy after unilateral ovariectomy (Trkulja et al., 2004). While we are not aware of the functional roles of ovarian M3 or M5, Cruz et al. previously described the presence of M1 receptors associated with follicular structures (Cruz et al., 2015). They reported M1 immunoreactivity localized mainly in theca cells and to a small extend in corpora lutea. Other studies suggested that the stimulation of these receptors either with ACh or with a muscarinic agonist could regulate progesterone secretion from the ovary (Delsouc, Della Vedova, et al., 2016; Delsouc, Morales, et al., 2016). Orozco et al. (2010) described that ACh in the celiac ganglion ovary preparation inhibits progesterone secretion from the ovary but stimulates testosterone secretion from the ovary in vitro. This suggests that the effect of ACh could be the result of ACh acting on M1 receptors, although located at the cell bodies of the postganglionic sympathetic neurons, rather than within the ovary.

In the present study, we combined immunohistochemistry, mRNA detection, and western blot analyses to investigate their presence and localization. Our results show that the rat ovary contains M1, which is present on GCs from primary to antral follicles and is localized to luteal cells. We do not have a full explanation for the discrepancy regarding the localization of the M1 receptor between our present data and the one mentioned above by Cruz et al (2015). Possibly, differences in the strain of the rats used (Sprague–Dawley in our study versus a CIIZ‐V strain in the study by Cruz et al.) are involved. Furthermore, in contrast to our study, Cruz et al. did not perform preadsorption studies, and importantly, they used a different antibody (sc‐9106, Santa Cruz Biotechnology versus our validated antibody from Alomone Lab). Indeed, antibody specificity is crucial as the specificities of many antibodies were questioned (Rommel et al., 2015).

While we did find only traces of M3 in the rat ovary, we detected next to M1 and also the M5 subtype. Previous reports had described the M5 gene by RT‐PCR in human granulosa cells (Fritz et al., 2001). The special M5 localization, almost exclusively associated with blood vessels surrounding the follicles, raises the possibility that M5 could be involved in the control of blood flow in the ovary. In other tissues, ACh modifies the vascular tone by regulating the local production of nitric oxide (NO) (Delsouc, Della Vedova, et al., 2016; Kozlowska et al., 2014; Uchida, 2014). Such a situation may also exist in the rat ovary and M5 activation could mediate, among others, the steroidogenic response by regulating blood flow. Such a possibility is based on reports, which described an antiestrogenic effect of NO locally applied to the ovarian compartment in an in vitro system (Delsouc, Morales, et al., 2016). Another study showed that estradiol not only increased the cholinergic neurons markers but also the enzyme that synthesizes NO (Jana et al., 2018). Thus, a possible role of M5 receptors in the rat ovary could be related to blood flow and hence indirectly to steroid efflux from the ovary.

Overall, the results of our study indicate that the rat ovary presents two main subtypes of MR, namely M1 and M5. M3 appeared only marginally detectable by our methods. Of note, the other MRs, M2 and M4, were not examined here.

We performed also two types of denervation studies of the rat ovary and the results showed that while NE was clearly decreased, ovarian ACh was not, strongly arguing for a local production of ACh. This is most likely located in the follicular compartments of the ovary (Urra et al., 2016). Hence, the sites of synthesis and MR, that is, the sites of actions of ACh are close together within the same ovarian compartments.

Sympathetic stress is a condition characterized by increased levels of intraovarian ACh (Riquelme et al., 2019), which as we found, however, did not change mRNA or M1/M5 proteins levels. Hence, increased ACh did not “downregulate” these receptors, as expected in a neuronal setting.

Some pieces of evidence exist regarding cholinergic stimulation of ovarian functions, although at the hypothalamic level (Cruz et al., 2015) and at the ganglionic region of the mesenteric territory. In these cases, the M1 blocker pirenzepine inhibited LH and hence ovulation. In the same study, the authors did not find changes in progesterone or estradiol plasma levels. It cannot be ruled out that upon intraovarian administration of an M1 blocker, this drug could diffuse away from the ovary and act in the central nervous system or that it acts through a sensory pathway to regulate the LH release from the pituitary by an action at the hypothalamus and hence GnRH secretion (Orozco et al., 2010). The administration of ACh to an ex‐vivo superior mesenteric ganglion—ovarian plexus nerve—ovary preparation, decreased progesterone and increased androstenedione in parallel to an increase in NO, suggesting an indirect action of ACh on steroid secretion from the ovary (Delsouc et al., 2019). Whether ACh may act, however, locally within the ovary and could regulate steroid production by activating MR of, for example, GCs remained unknown. We addressed this question but did not find evidence for such a stimulatory effect. We used the muscarinic agonist carbachol and tested steroid secretion in an ovarian incubation system. This in vitro system employed was previously successfully used to study the local effect of gonadotropins, beta‐adrenergic agonists, or other peptides from the ovary (Barria et al., 1993; Squicciarini et al., 2018). Hence, method‐related problems are unlikely. Furthermore, the lack of regulatory action on steroid production was supported by the lack of an effect of carbachol and HupA on expression levels of the enzymes participating in the biosynthesis of steroids.

Finally, we designed and executed a study to investigate the effect of a muscarinic antagonist (atropine) applied locally to the ovary for 28 days. This long‐term experiment was based on the fact that a cohort of follicles takes at least 4 weeks to reach ovulation (Greenwald & Roy, 1994). As found in the in vitro experiments, we did not find changes in the plasma levels of ovarian steroids either. Yet, there were clear changes in the development of the follicular cohort. Atropine increased the presence of atretic follicles. Studies in the ovary of the rat (Urra et al., 2016), macaque, or in human granulosa cells (Mayerhofer & Fritz, 2002) indicate that ACh can act on GCs. These actions may also occur during the first steps of follicular development, that is, when M1 is detectable in the rat. We, therefore, suggest that in rat ovary, ACh, presumably acting through M1, is likely responsible for changes in early follicular development. Conversely, atropine interferes with this growth‐promoting action and consequently causes GC death and follicular atresia.

In summary, the results provide novel details about ovarian ACh and its roles in the rat. ACh levels do not change after ovarian denervation, indicating that the majority of ovarian ACh has intra‐ovarian sources. Increased atresia after in vivo‐blockage of MR by atropine indicates that ovarian ACh exerts trophic effects via MRs. While the possible involvement of M2/M4 remains to be established, M1 of GCs may be involved. Additional ACh from vagal nerve origin might act on ovarian blood flow through M5. Altogether these data give strong support for a physiological role of ACh in ovary function, the fact that we could regulate blood flow through different receptors (probably M5) and the activation of follicular development (through M1), open unsuspected possibilities to regulate ovary function by using agonist/antagonist to the subtypes of muscarinic receptors to act specifically in some of the multiples targets regulating the ovary function. Additional studies are required to complement the knowledge about the ovarian cholinergic system of the rat ovary.

## AUTHOR CONTRIBUTIONS

Fernanda C. Cuevas performed most of the experimental work with rats, biochemical analysis, data collection, and manuscript preparation. Pia Sessenhausen studied the effect of Carbachol and Hup‐A on the expression of steroidogenic enzymes. Daniela Bastias and Constanza Alanis performed the morphometric analysis of atropine experiments. Agustin Benitez did the measurements of NE and ACh for denervation studies. Raul Riquelme and Valentina Squicciarini did the PCR and WB analyses. Hernan E. Lara and Artur Mayerhofer conceived the idea, participated in the study design, and directed the work and manuscript preparation. All authors participated in writing and approved the final version of the manuscript.

## FUNDING INFORMATION

This study was supported by grants from Fondo Nacional de Ciencias Fondecyt 1170291 (to HEL) and DFG Project number 432434245 (to AM). FC was also supported by a scholarship for Doctoral thesis support Conicyt No. 21161032. This work was performed in partial fulfillment of the requirements of a PhD degree in pharmacology to FC and of a Dr. rer nat. degree of PS, respectively.

## CONFLICT OF INTEREST

The authors declare that there is no conflict of interest that could be perceived as prejudicing the impartiality of the research reported.
